# Patterned polymer matrix promotes stemness and cell-cell interaction of adult stem cells

**DOI:** 10.1186/s13036-015-0016-x

**Published:** 2015-10-12

**Authors:** Lucas H. Hofmeister, Lino Costa, Daniel A. Balikov, Spencer W. Crowder, Alexander Terekhov, Hak-Joon Sung, William H. Hofmeister

**Affiliations:** Department of Biomedical Engineering, Vanderbilt University, Nashville, TN USA; Center for Laser Applications, University of Tennessee Space Institute, Tullahoma, TN USA

**Keywords:** Stem cell, Matrix, Polycaprolactone, Nanopore, Nanofiber

## Abstract

**Background:**

The interaction of stem cells with their culture substrates is critical in controlling their fate and function. Declining stemness of adult-derived human mesenchymal stem cells (hMSCs) during *in vitro* expansion on tissue culture polystyrene (TCPS) severely limits their therapeutic efficacy prior to cell transplantation into damaged tissues. Thus, various formats of natural and synthetic materials have been manipulated in attempts to reproduce *in vivo* matrix environments in which hMSCs reside.

**Results:**

We developed a series of patterned polymer matrices for cell culture by hot-pressing poly(ε-caprolactone) (PCL) films in femtosecond laser-ablated nanopore molds, forming nanofibers on flat PCL substrates. hMSCs cultured on these PCL fiber matrices significantly increased expression of critical self-renewal factors, Nanog and OCT4A, as well as markers of cell-cell interaction PECAM and ITGA2. The results suggest the patterned polymer fiber matrix is a promising model to maintain the stemness of adult hMSCs.

**Conclusion:**

This approach meets the need for scalable, highly repeatable, and tuneable models that mimic extracellular matrix features that signal for maintenance of hMSC stemness.

## Background

The ability to manipulate expansion and differentiation of stem cells has been on the forefront of materials research for several decades. However, many synthetic culture substrates resulting from these research efforts embody properties that are not completely biomimetic. In response, the biomaterials community has been reorienting their design process towards generating an optimal stem cell culture substrate that can reliably and reproducibly mimic the extracellular matrix (ECM), one of the primary environmental constituents that heavily influences cell behavior [1]. Generally, altered mechanical and chemical properties of cell substrates have been shown to alter tissue homeostasis, stem cell differentiation, and metastasis, as examples [2]. All of these physiological responses are initiated by “outside-in” signaling, where physical cues regulate cell function. For stem cells, and human mesenchymal stem cells (hMSCs) in particular, design parameters for improved *in vitro* culture systems can heavily influence their decision to either maintain their stem cell phenotype or differentiate towards a specified cell lineage.

Guided by physical cues incorporated into the substrate design, numerous example culture systems have been and continue to be explored for diverse cell culture purposes. Poly(ε-caprolactone) (PCL), a common synthetic polymer used in biomedical applications, has been shown to help push hMSCs towards neural lineages when the material’s hydrophobic nature is coupled with soluble factor neural-induction media [3]. Synthetic copolymers, such as poly(vinyl alcohol)-PCL, create 3D structures reminiscent of native tissues and when loaded with growth factors have promoted hMSC differentiation into chondrocytes, thereby generating structurally robust cartilage tissue [4]. Combining polymers with metals and minerals have also shown promise in encouraging MSCs to create complex layered tissue structures such as bone-cartilage interfaces [5] in addition to providing templates to investigate MSC responses to high-throughput chemical screening protocols [6]. Finally, natural polymers like collagen have also been modified with peptides to encourage better tissue regeneration by jump-starting growth responses in slow proliferating cells like dorsal root ganglion cells [7].

Considering the importance of both cell-cell and cell-matrix adhesion molecule interactions in maintaining hMSC stemness [8–11], it is clear that flat substrates like tissue culture polystyrene (TCPS) cannot fully recapitulate the native hMSC environment (e.g. bone marrow) that balances these two types of binding events. As a result, mechanisms of self-organization, endogenous matrix deposition, differentiation, and remodeling that encompass the traditional hMSC phenotype are disturbed [12]. Additionally, the minimal flexibility of modifying cell-adhesive surfaces of existing mass-produced culture platforms complicates the ability to probe and understand stem cell behavior as it relates developmental and regenerative processes [13, 14]. This obstacle has, thus, spurred the recent explosion in the use of gel culture systems such as polyacrylamide hydrogels [15–18], collagen hydrogels [19, 20], and Matrigel® [21].

Despite the contributions made by the research community, these materials do have limitations. First, it is difficult to uncouple gel properties, which limits the ability to engineer controlled cellular responses to isolated stimuli. For example, changing pore size alters gel rigidity and fiber architecture that may result in substrate properties unrepresentative of native hMSC-containing tissues [22]. Chemical transport is also inhibited across the boundary of the gel, which could produce shortfalls of chemokines and similar molecules that help hMSCs maintain their naïve phenotypes [23]. Furthermore, batch-to-batch inconsistency can obfuscate fundamental mechanisms being studied that pertain to hMSC homeostasis [24]. Given the challenges with gel systems, other research groups have turned to electrospinning as another potential approach to generate synthetic cell culture models [25]. Electrospinning is limited by the challenges of variations in fiber morphology and internal void structure due to the complexity of the fabrication process. Hence, with all the aforementioned shortfalls of existing biomaterials approaches, the development of scalable and physiologically relevant biomimetic culture models that mimic hMSC niches to maintain stemness *in vitro* remains a top priority as very few studies have been able to design culture templates that successfully achieve this goal [26].

To overcome some of these challenges, we created biomimetic substrates with hierarchical architecture by hot-pressing PCL into patterned laser-ablated nanopore molds. When extracted, these PCL substrates consist of polymer nanofibers patterned on the micron scale over square centimeters of culture substrate surface. The structures are similar in size and morphology to collagen fibrils found within the bone marrow *in vivo* [20, 24, 27]. The attachment of fibers at the substrate base mimics the basement membrane where collagen fibrils are in contact with a highly cross-linked collagen IV layer [28]. Altering the nanopore molds can control the spacing, length, diameter, and pattern of the polymer fiber matrix. Collectively, this molding method provides reproducible substrates that eliminate variability and precisely control fiber topography. When used to culture hMSCs, these polymer fiber models were found to significantly increase expression of critical regulators of self-renewal, as well as markers indicative of increased cell-cell interaction that are paramount in stem cell homeostasis [8–11].

## Results and discussion

### Polymer fiber substrate fabrication

Recently, Rajput and co-workers [29] demonstrated a novel and simple process of fabricating polymer films covered with large arrays of standing polymer fibers that mimic the fibrillar environment found in the extracellular matrix. In this process, a polymer-solvent solution is cast on the surface of a fused silica mold where an array of ultra-high aspect ratio surface nanopores is formed via the femtosecond laser ablation method first described by White et al. [30]. The polymer-solvent solution fills the surface nanopores through capillary action, and as the solvent dissipates, polymer fibers form within these nanopores. Once the solvent evaporates completely, the resulting polymer film is gently peeled-off the surface of the fused silica chip, producing an array of standing polymer fibers.

**Fig. 1:**
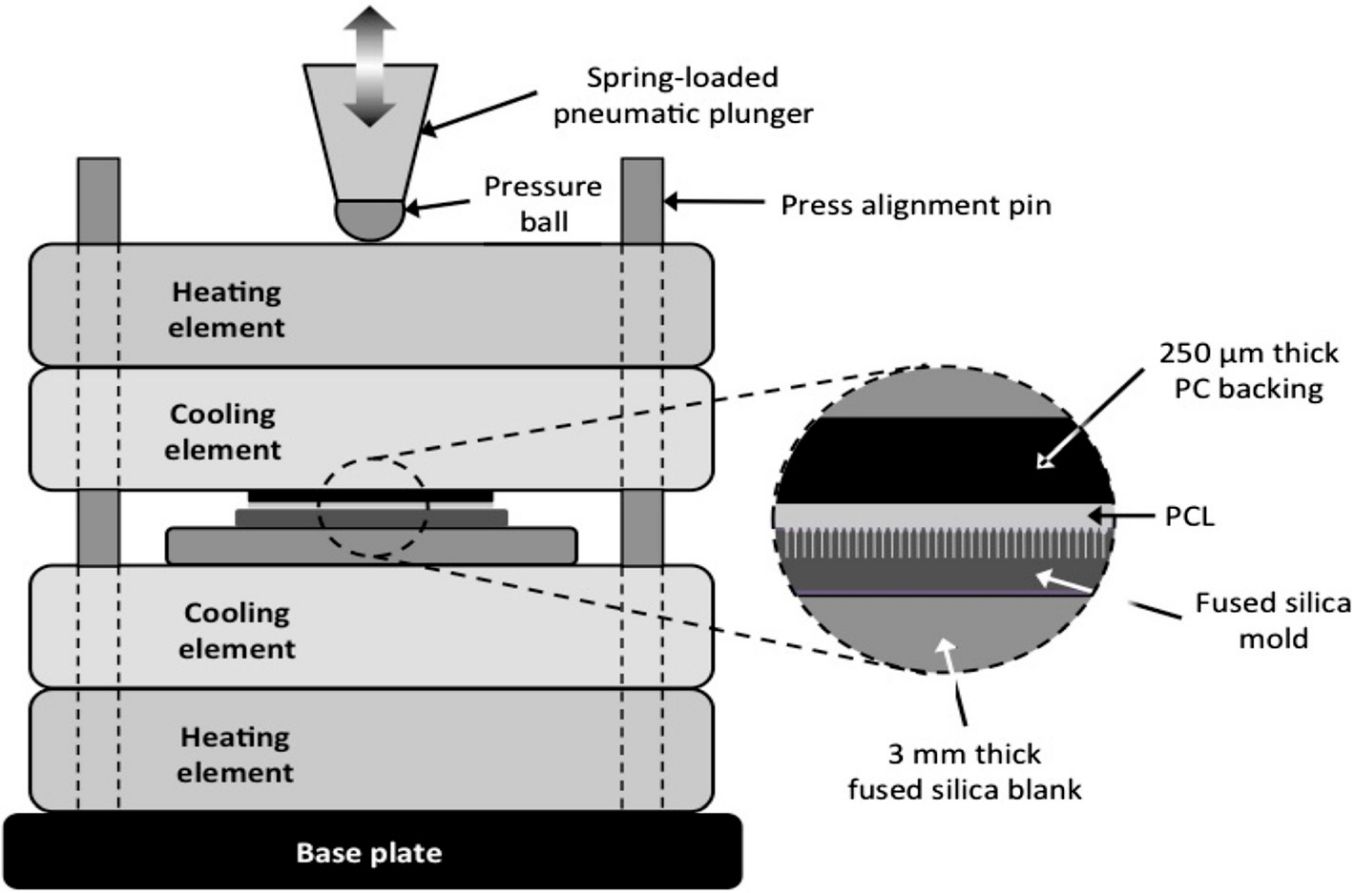
Basic components and layout of the hot-pressing system

In the present study we fabricated nanofibers using a new technique of hot-pressing, a solvent-free process applicable to thermoplastic polymers. The polymer film and the fused silica mold are pressed against each other and warmed above the polymer melting temperature for 5 minutes, allowing molten polymer to flow into the nanopores of the mold surface. Once the materials return to room temperature, the resulting nanofiber polymer film is peeled off the mold. This process is much faster than solvent casting and yields more fully formed fibers with fewer defects than the casting process. Hot pressing eliminates the use of solvents which in trace amounts can affect cell fate. A wider range of chemical dopants can also be used when polymer solvents are eliminated. Ultra-high aspect ratio nanopore molds can be machined with any pattern of holes or lines with the femtosecond laser to generate the nanofiber arrays with complex geometries [31–34]. Table 1 lists the various layouts used for this study, and the basic components of a hot-pressing system are shown in Fig. 1. As ablated, the nanopores have diameters as small as 50 nm at the bottom and entrance holes as small as 150 nm. The diameter and depth of the nanopores can be adjusted by varying the focus depth and laser energy per laser pulse. Nanopores can be etched in hot KOH to further enlarge the diameters up to 1 μm. The hydroxide has high specificity (>100:1) for laser-damaged silica, which occurs in the diffraction-limited focal spot surrounding the nanopore. With these parameters of fabrication, the reusable fused silica molds can be prepared with over 25 million nanopores per square centimeter.

**Table 1:** Mold identification with process times and resulting polymer fiber measurement statistics

Silica Mould ID	Pore spacing X axis (μm)	Pore spacing Y axis (μm)	Nanopores per area (100 μm^2^)	Laser Energy per pulse (μJ)	KOH etch time, molarity (hours, Molarity)	Fiber width mean ± SEM (μm)	Fiber height mean ± SEM (μm)
2 × 2	2	2	25	1.4	1, 10 M	0.15 ± 0.03	30.0 ± 5.0
2 × 3	2	3	16.7	2	1, 10 M	0.29 ± 0.05	25.0 ± 10.0
4 × 4	4	4	6.2	2	1, 10 M	0.27 ± 0.05	30.0 ± 5.0
5 × 5	5	5	4	4	3, 5 M	0.46 ± 0.05	30.0 ± 5.0
7 × 7	7	7	2	1.8	2, 10 M	1.10 ± 0.08	25.0 ± 0.5
8 × 8	8	8	1.5	1.8	2, 10 M	0.91 ± 0.07	16.0 ± 0.3
10 × 10	10	10	1	2	2, 10 M	0.92 ± 0.07	24.0 ± 1.0

**Fig. 2:**
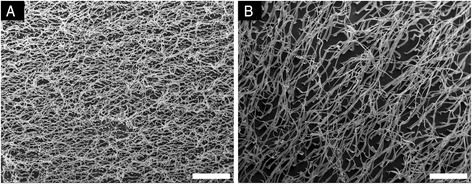
Scanning electron micrographs of two small fiber models taken parallel to the substrate at the same magnification. **a** The 2 × 2 pattern has the smaller diameter fibers and the highest density of fibers at 25 per 100 μm^2^. **b** The 5 × 5 mold has larger diameter fibers and a density of 4 fibers per 100 μm^2^. Scale bars are 20 μm

**Fig. 3:**
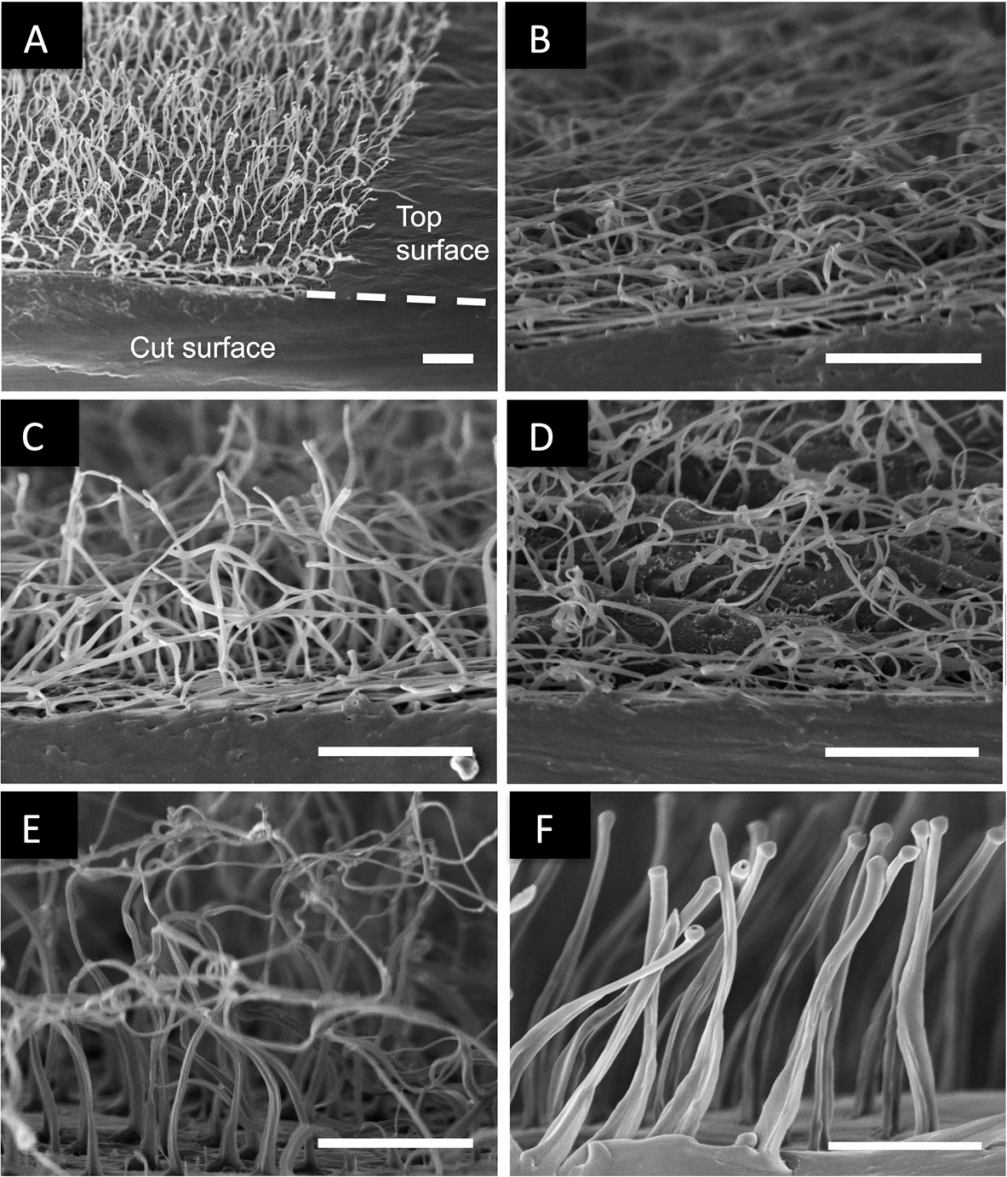
Transverse sections of freestanding polymer fiber films. **a** Polymer fiber substrate overview showing the cut surface for transverse sections. **b** 2 × 2 polymer fiber substrate. **c** 2 × 3 polymer fiber substrate. **d** 4 × 4 polymer fiber substrate. **e** 5 × 5 polymer fiber substrate. **f** 7 × 7 polymer fiber substrate. Scale bars are 10 μm

Long polymer fibers can be formed in and extracted from high aspect ratio nanopores via casting, previously mentioned, or hot-pressing as described in this study. SEM images of typical arrays of PCL polymer fibers formed by hot-pressing are shown in Fig. 2. Transverse sections of the fiber models are shown in Fig. 3. Average diameters and height of the fiber mat are given in Table 1. Polymer fiber morphology depends on etch time. In all conditions, fiber structures were observed to be wider at the base than at the tip, similar to the nanopore morphology reported previously [29]. Substrates formed using 2 × 2, 2 × 3, 4 × 4, and 5 × 5 molds had average fiber diameters under 500 nm and average fiber lengths greater than 30 μm. Substrates formed using 7 × 7, 8 × 8, and 10 × 10 molds yielded fibers with average diameters around 1 μm and lengths between 15 and 25 μm. Employing two-hour etch times resulted in larger pore size and larger fiber diameters. Polymer fibers in the 2 × 2, 2 × 3, 4 × 4, and 5 × 5 did exhibit some stretching of the polymer fibers during removal from the mold, but these deformations were not significant such that consistency in fabrication was compromised. Overall, these results demonstrate the tunability of fiber morphology using this technique.

### hMSC response to polymer fiber models

hMSCs were cultured on flat or polymer nanofiber substrates for 96 h in order to allow for the cells to completely acclimate to their culture substrate, which includes recognizing the presence of the substrate, receiving the outside-in material cues, altering cell gene and protein expression, and implementing the new cell morphology/tissue structure. The substrates themselves did not degrade during the culture period and the polymer nanofibers could still be seen by brightfield microscopy. The integrity of the polymer substrates was also maintained during the media change and no media leakage was observed between the PCL and the polycarbonate backing.

**Fig. 4:**
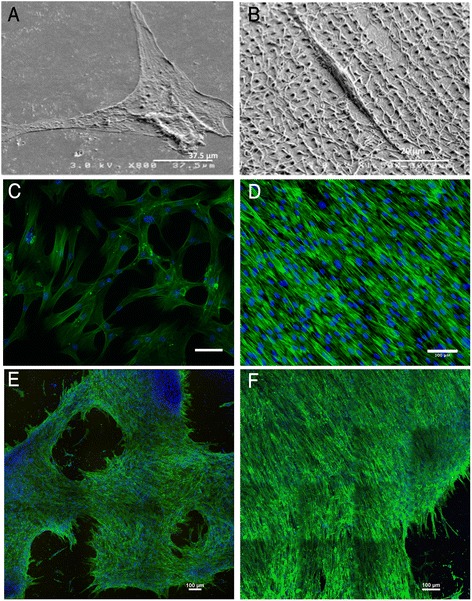
hMSC responses to polymer models. Optical and SEM images of hMSCs on culture models. **a** SEM on flat PCL. **b** SEM on 10 × 10 polymer fibers. **c** Optical on flat PCL. **d** Optical on 2 × 2 polymer fibers. **e** Optical on 2 × 3 polymer fibers. **f** Optical on 10 × 10 polymer fibers. Green = F-actin, Blue = nucleus. Scale bars in fluorescent images are 100 μm

On the flat PCL substrates, we observed prominent hMSC spreading morphology with large, wide membrane protrusions that maximized cell membrane surface contact area with the polymer (Fig. 4a & c). These cells also demonstrated non-specific organization of their actin cytoskeleton (green) (Fig. 4c). When cultured on polymer nanofiber substrates, hMSCs were observed to interact directly with the polymer nanofibers (Fig. 4b) and were strictly oriented along rows of fibers with a more spindle shape morphology. This observation has been well documented with micro-patterned substrates in numerous other studies and is termed “contact-guidance” [35]. Only when cultured on polymer nanofibers were hMSCs seen to organize into tissue-like morphologies, complete with alignment of actin cytoskeleton (green) (Fig. 4d). Interestingly, large-scale tissue-like structures were regularly observed on polymer nanofiber models regardless of spacing (Fig. 4e & f).

### Gene expression analysis

**Fig. 5:**
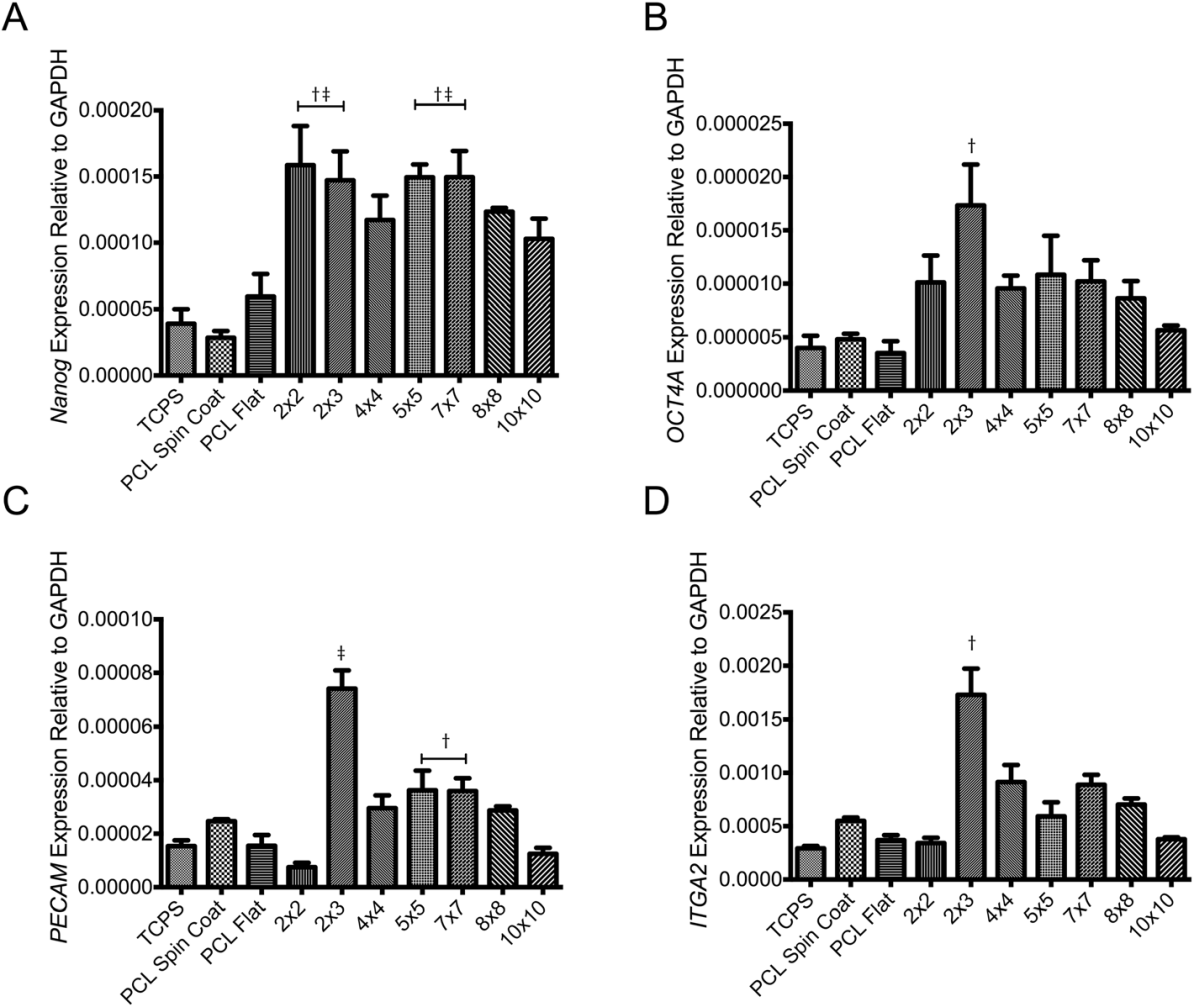
Gene expression on polymer fiber substrates. **a** Nanog expression relative to GAPDH. † Indicates *p* < 0.05 relative to TCPS. ‡ Indicates *p* < 0.05 relative to PCL spin coat. **b** OCT4a expression relative to GAPDH. † Indicates *p* < 0.05 relative to TCPS. **c** PECAM expression relative to GAPDH. † Indicates *p* < 0.05 relative to 4 × 4. ‡ Indicates *p* < 0.05 relative to all other groups. **d** ITGA2 expression relative to GAPDH. † Indicates *p* < 0.05 relative to all other groups

When cultured on polymer nanofiber substrates for 4 days, hMSCs significantly increased expression of the transcription factors *Nanog* and *OCT4A*, which are critical for self-renewal of stem cells [36]. This increase in gene expression was most significant on 2 × 3 models, but remained increased over TCPS and flat PCL controls for all polymer fiber templates (Fig. 5a & b). These findings also appear to positively correlate with the presence of tissue-like structures that formed only on the polymer nanofiber substrates. With respect to cell adhesion interactions, hMSCs exhibited significantly higher cell-cell interactions and lower cell-matrix interactions compared to cells on the flat substrates (Fig. 5c & d). These results are visually supported by the morphologies of hMSCs cultured on polymer nanofiber and flat models (Fig. 4). The significant increase in expression of platelet endothelial cell adhesion molecule 1 (PECAM), an indicative marker of cell-cell interaction, peaked on the 2 × 3 model (Fig. 5c) [37, 38]. In addition, culture on polymer nanofiber substrates increased the expression of integrin subunit alpha 2 (ITGA2), which also is indicative of increased cell-cell interaction (Fig. 5d) [39].

Many studies investigating basic MSC physiology utilize “hanging drop” models as this culture platform has been reported as the best available bone marrow-analog culture model in a 3D format [40]. MSCs in this setting secrete their own matrix to allow for cell-matrix adhesion events, but the additional presence of cell-cell adhesion events given the substrate-less format of the hanging drop model appears to be mimicked by the polymer nanofiber substrates. As such, the 2 × 3 model likely provided the same balance of available cell-matrix adhesion by the surface area of the nanofibers themselves, covering any flat PCL underneath the fibers that would overcome potential cell-cell adhesion events known to occur among hMSCs. In conclusion, these results indicate that varying polymer fiber spacing can alter cell-cell and cell-matrix interactions.

## Conclusion

Here, we present the fabrication and implementation of new polymer nanofiber cell culture substrates for hMSCs. We demonstrated the tunability of the polymer nanofiber models by varying the template nanopore spacing and etch time. When hMSCs were cultured on these polymer nanofiber models compared to TCPS or flat PCL substrates, their stemness was significantly improved likely by promoting cell-cell over cell-matrix interactions. The inherent ability of the polymer models to promote contact guidance of hMSCs lead to large scale coordinated behavior and ultimately the formation of tissue like structures [41]. We observed hMSCs interaction directly with polymer fibers, which resulted in gross morphological differences between cells cultured on flat substrates. These morphological changes were accompanied by significant increases in expression of key self-renewal factors *Nanog* and *OCT4A*, as well as significantly increased expression of cell-cell interaction markers *PECAM* and *ITGA2*. Of the groups tested, the 2 × 3 polymer nanofibers showed the most drastic increases in stemness and cell-cell interactions. From this result, we hypothesize that the increase in expression of self-renewal factors was mediated by increased cell-cell interaction that could only occur if the culture substrate embodied characteristics native to the bone marrow environment. Future studies utilizing these templates can work towards understanding the signaling mechanism linking increased cell-cell adhesion with increased stemness gene expression.

Taken together, these polymer nanofiber substrates serve as ECM mimetic substrates that can reinstate hMSC stemness. With increased expression of stemness and cell-cell marker genes, certain fiber arrangements promote a pseudo status of “forced aggregation” in hMSC culture. In contrast to traditional three-dimensional substrates, these polymer models provide drastic improvements in terms of consistency, ease of use, tunability, and scalability while at the same time providing one of the few culture substrate options that allow access to hMSCs that have not lost their stemness expression in *in vitro* culture.

## Materials and methods

### Materials

All reagents were purchased from Sigma-Aldrich (St. Louis, MO) at the highest available quality unless otherwise noted.

### Culture substrate fabrication

For this work, a one millimeter thick, double side polished fused silica wafer (Mark Optics, Santa Ana, CA) was diced into 22 mm by 22 mm square chips. Each chip was patterned with a single 10 mm by 10 mm array of nanopores using the femtosecond laser machining system [29]. Each array was patterned with a unique pore-to-pore spacing value, determined by the choice of laser beam raster, laser pulse repetition rate and laser beam scanning rate. Each nanopore was formed by a single 790 nm wavelength, 160 femtosecond laser pulse focused on the surface of the fused silica chip using a dry microscope objective (Nikon CF Plan Achromat 79173) with a numerical aperture (NA) of 0.85 and spherical aberration correction collar set to 0.17 mm. The as-processed fused silica molds were soaked in aqueous KOH at 90 °C to remove femtosecond laser ablation debris and also to enlarge the diameter of the nanopores. The etched molds were rinsed then soaked in deionized water at 90 °C for 2 h to remove residual KOH, and finally dried under a stream of dry nitrogen.

To facilitate the release of polymer fibers from the mold, all fused silica molds were silanized with 1H1H2H2H Perfluorodecyltrichlorosilane (FDTS) (Alfa Aesar, Ward Hill, MA). First the molds were conditioned in a 1:1 mix of HCl:methanol for 30 min. The molds were then rinsed in methanol, dried under a stream of dry nitrogen, and finally exposed to FDTS vapors inside a 200 millitorr desiccator for 12 h. FTDS molecules bind to –OH terminated surfaces and form self-assembled monolayers that reduce surface energy and prevent sticking.

Each mold listed in Table 1 was used to prepare PCL nanofiber culture models via hot-pressing. A piece of PCL film, formed by compression of PCL pellets, is placed between a fused silica mold and a 22 mm by 22 mm by 0.25 mm HybriSlip HS22-CS polycarbonate (PC) backing slide (Grace Bio-labs, Bend, OR). This three-element stack is prepared atop a 3 mm thick fused silica flat blank seated at the center of the press (Fig. 1). With all the elements of the press stacked together, the spring-loaded pneumatic plunger is actuated progressively to a pressure of 45 psi, pressing the pressure ball against the stack. The Chrome-Nickel heating elements inside the heating blocks are turned on, and the temperature of the cooling elements (monitored using a pair of thermocouples) is raised to and held at 80 °C for 5 min. During this period, the PCL melts and infiltrates the nanopores of the mold. The heating elements are then turned off, and the press allowed to cool to 50 °C by air convection. Once the temperature of the cooling elements reaches 50 °C, rapid cooling to room temperature is forced by circulating cold water through the cooling elements. Once room temperature is reached, the plunger is allowed to pull back to its idle position. The mold-PCL-PC stack is removed from the press, and PCL-PC is gently peeled-off the mold. The PCL adheres strongly to the PC backing, making it easy to handle.

Flat PCL substrates were formed by the press apparatus, and spin-coated control-substrates were formed by a spin-coating apparatus (Laurell Technologies, North Wales, PA, USA). For spin-coated substrates 15 mm circular glass cover slips (Fisher Scientific) were first cleaned with 100 % ethanol, rinsed with deionized water, and heated to 80 °C for ~20 min to dry. A 1 % weight/volume (w/v) solution of PCL in tetrahydrofuran (THF) was spun for 30 s at 3000 RPM atop the clean glass cover slip (50 μl polymer solution/sample).

### Substrate characterization

For scanning electron microscopy (SEM) imaging of polymer fibers, we used JEOL JSM-6320 F scanning electron microscope (JEOL, Tokyo, Japan). Samples for SEM imaging were prepared by cross sectioning PCL on PC substrates with a razor blade. To prevent PCL films from charging during SEM imaging, every sample was sputter-coated with a 20–30 nm thick gold film using a Bio-Rad Polaron SEM coating system E5150 with film thickness control (Quorum Technologies, UK). Polymer fiber dimensions were measured using ImageJ (NIH, Bethesda, MD). During the mold fabrication process, the laser pulse creates an entrance hole in the mold that has a larger diameter than the majority of the hole. This results in a wider base on each polymer fiber that is 1–2 μm in height. These bases were excluded from the fiber diameter measurement. Fifteen diameter measurements were performed on two images per each polymer fiber mold. These measurements include the sputter coating thickness. The height of fibers above the base was measured by optical microscopy. A 50x objective was focused, at first, on the substrate, and then translated vertically until the tops of the fibers were in focus. The translation distance was measured on a micrometer. Focus was verified using brightfield and darkfield functions. Optical height measurements are consistent with SEM imaging.

### Cell culture

hMSCs were purchased from Lonza (Walkersville, MD). All cell experiments used hMSCs at passage 5. hMSCs were cultured in alpha-minimum essential media with nucleosides (Life Technologies, Carlsbad, CA), 16.7 % heat-inactivated fetal bovine serum (Life Technologies), 1 % penicillin/streptomycin (Life Technologies), and 4 μg/ml plasmocin prophylactic agent (InvivoGen, San Diego, CA). Cells were grown in a humidified incubator at 37 °C and 5 % CO_2_. Media was replaced every 3 days. hMSCs, were detached from tissue culture flasks at around 80 % confluence with 0.05 % trypsin-EDTA and passaged at 100–500 cells/cm^2^. For all cell experiments, hMSCs were seeded on substrates at a density of 10,000 viable cells/cm^2^. Cell media was replaced after 72 h.

### Quantitative real-time polymerase chain reaction (qPCR)

Cells cultured on polymer fiber films and TCPS control wells were homogenized with Trizol reagent (Life Technolgies), mixed with chloroform (1:5 Trizol:chloroform), and separated by centrifugation (12,000x g, 15 min, 4 °C). The aqueous phase containing RNA was isolated using RNeasy columns (Bio-Rad, Hercules, CA) according to the manufacturer’s instructions. RNA concentration was determined using a TECAN M1000 plate reader with the manufacturer’s software. cDNA was synthesized using a cDNA generation kit (Applied Biosystems, Life Technologies, Carlsbad, CA) and qPCR was performed using SYBR Green master mix kit (Bio-Rad) with 15 ng cDNA and 500 mM each of forward and reverse primers. Primer sequences were the following: Nanog (NM_024865.2) forward ‘AATACCTCAGCCTCCAGCAGATG’ and reverse ‘TGCGTCACACCATTGCTATTCTTC’; OCT4A (NM_002701.4) forward ‘CCTTCGCAAGCCCTCATTTCAC’ and reverse ‘GGAAGCTTAGCCAGGTCCGA’; ITGA2 (NM_002203.3 forward ‘TTAGCGCTCAGTCAAGGCAT’ and reverse ‘CGGTTCTCAGGAAAGCCACT’; PECAM (NM_00442.4) forward ‘CCAAGCCCGAACTGGAATCT’ and reverse ‘CACTGTCCGACTTTGAGGCT’; and GAPDH (NM_002046.4) forward ‘GCACCGTCAAGGCTGAGAAC’ and reverse ‘TGGTGAAGACGCCAGTGGA’. A CFX Real-Time PCR System (Bio-Rad) was run with the qPCR protocol: 95 °C for 3 min, followed by 40 cycles of denaturation at 95 °C for 30 s, annealing at 58 °C for 30 s, and extension at 72 °C for 30 s. Expression of each gene measured was normalized to the expression of glyceraldehyde 3-phosphate dehydrogenase (GAPDH) as a housekeeping gene, thereby generating ΔC(t) values, and expression of 2^-ΔΔC(t)^ relative to the TCPS control. *N* = 3 biological replicates per substrate condition were performed.

### Immunocytochemistry

Cells cultured on the test substrates were fixed with 4 % paraformaldehyde (PFA) for 15 min at room temperature and permeabilized with 10 % goat serum with 0.3 % Triton-X overnight at 4 °C. Cells were then incubated with Hoechst (2 μg/ml) for 20 min at room temperature, followed by Alexa488-phallodin (1:5 v/v in PBS, Life Technologies, Carlsbad, CA) for 10 min. Imaging was performed with a Zeiss LSM 710 confocal microscope (Carl Zeiss, Oberkochen, Germany) and images were process with Zeiss Zen software and ImageJ (NIH, Bethesda, MD).

## Abbreviations

hMSCs: human mesenchymal stem cells
TCPS: Tissue culture polymer styrene
PCL: Poly(ε-caprolactone)
ECM: Extracellular matrix
SEM: Scanning electron microscopy
ITGA2: Integrin-α2
PECAM: Platelet endothelial cell adhesion molecule 1
OCT4A: Octomer-binding transcription factor 4A
NANOG: Homeobox protein Nanog
